# Application value of coaxial puncture needle (technique) in ultrasound-guided puncture biopsy of peripheral pulmonary masses

**DOI:** 10.1097/MD.0000000000031070

**Published:** 2022-11-11

**Authors:** Mei Wu Zhang, Yan Zhang, Shu Yi Lv, Xiao Xiang Fan, Jia Zhen Zhu, Bai Song Zhang, Zhen Hua Yang

**Affiliations:** a Department of Interventional Therapy, Hwa Mei Hospital, University of Chinese Academy of Sciences, Ningbo, Zhejiang, P.R. China; b Ningbo Clinical research Center for Medical Imaging, Ningbo, Zhejiang, P.R. China; c Provinicial and Municipal Co-construction Key Discipline for Medical Imaging, Ningbo, Zhejiang, P.R. China.

**Keywords:** coaxial puncture, lung biopsy, peripheral lung nodules, ultrasound guidance

## Abstract

This study aims to investigate the effect of ultrasound (US)-guided coaxial puncture needle in puncture biopsy of peripheral pulmonary masses. In this retrospective analysis, 157 patients who underwent US-guided percutaneous lung biopsy in our hospital were divided into a coaxial biopsy group and a conventional biopsy group (the control group) according to the puncture tools involved, with 73 and 84 patients, respectively. The average puncture time, number of sampling, sampling satisfaction rate, puncture success rate and complication rate between the 2 groups were compared and discussed in detail. One hundred fifty-seven patients underwent puncture biopsy, and 145 patients finally obtained definitive pathological results. The overall puncture success rate was 92.4% ([145/157]; with a puncture success rate of 97.3% [71/73] from the coaxial biopsy group and a puncture success rate of 88.1% [74/84] from the conventional biopsy group (*P* < .05). For peripheral pulmonary masses ≤3 cm, the average puncture time in the coaxial biopsy group was shorter than that in the conventional biopsy group, and the number of sampling, sampling satisfaction rate and puncture success rate were significantly higher than those in the conventional biopsy group (*P* < .05). There was no significant difference in the complication rate between the 2 groups (*P* > .05). For peripheral pulmonary masses >3 cm, the average puncture time in the coaxial biopsy group was still shorter than that in the conventional biopsy group (*P* < .05). The differences between the 2 groups in the number of sampling, satisfaction rate of the sampling, the success rate of puncture and the incidence of complications were not significant (*P* > .05). US guided coaxial puncture biopsy could save puncture time, increase the number of sampling, and improve the satisfaction rate of sampling and the success rate of puncture (especially for small lesions) by establishing a biopsy channel on the basis of the coaxial needle sheath. It provided reliable information for the diagnosis, differential diagnosis and individualized accurate treatment of lesions as well.

## 1. Introduction

Peripheral pulmonary masses, represented by lung cancer, can seriously affect patients’ health condition and life quality. With the development of technology, ultrasound (US) has been widely used in recent years.^[1]^ Owing to its convenience, zero-radiation, bedside operation and other advantages, US has been an emerging tool to guide myriad interventions.^[2]^ US-guided percutaneous lung biopsy, known as its excellent performance in disease diagnosis and staging, is a safe and effective minimally invasive technique, obtaining tissue samples from lesions to facilitate the differential diagnosis of primary tumors and metastatic tumors, infectious and non-infectious lesions.^[3,4]^ According to American Medical Insurance Administration, epidemiology and final results database, approximately 57% lung cancer patients have already developed into distant metastasis at initial diagnosis.^[5]^ These patients cannot be operated, and their follow-up treatment depends heavily on pathological diagnosis, including histopathological diagnosis, immunohistochemical staining and molecular pathological detection. All these examinations require biopsy to obtain specimens.

For peripheral pulmonary masses, US can indicate the internal structure and blood flow of most lesion. Thanks to its safe and radiation-free characteristics, and has the characteristics of multi-angle and multi-direction. Compared with computed tomography (CT), US-guided puncture is more convenient and fast.^[6]^ Thus, US-guided percutaneous biopsy is one of the most preferred methods of lung biopsy,^[7]^ and has been widely applied in clinic given that it rarely induces complications. However, in the past, the commonly used puncture biopsy needle (no matter semi-active puncture biopsy needle or automatic puncture biopsy needle), could only take 1 piece of tissue each time, which was utterly inefficient and sometimes even affected the follow-up punctures. With the unceasing medical technology progress, coaxial puncture technology has been gradually applied in CT guided lung puncture in recent years, obtaining multiple tissues in a fairly simple and practicable manner at one time, which is simple.^[6,8]^ In light of the fact that few reports on the application of coaxial puncture biopsy needle in US-guided pulmonary mass puncture have been published at home and abroad so far, this study is aimed to explore the application effect of the US-guided coaxial puncture needle in peripheral pulmonary mass puncture biopsy, especially for the pulmonary masses within 3 cm (i.e., ≤3 cm).

## 2. Materials and Methods

### 2.1. Patients

This study retrospectively collected the clinical data of patients with peripheral pulmonary masses who were hospitalized and underwent US-guided biopsy in Ningbo Hwa Mei Hospital of University of Chinese Academy of Sciences from January 2018 to February 2020. Inclusion criteria: patients with peripheral pulmonary masses and pleural lesions revealed by CT; patients who underwent voluntary US-guided biopsy.

Exclusion criteria: unclear lesions on US examination; patients with obvious bleeding tendency; those with cardiopulmonary insufficiency; those with severe cough or inability to cooperate with puncture.

A total of 157 patients were included. According to the puncture tools used, they were divided into a coaxial biopsy group and a conventional biopsy group, with 73 cases and 84 cases respectively. The study was approved by the Human Body Research Ethics Committee of Ningbo Hwa Mei Hospital, University of Chinese Academy of Sciences (YJ-KYSB-NBEY-2020-191-01). As this is a retrospective study, the patient’s informed consent was exempted.

### 2.2. Instruments and methods

Esaote My Lab90 color Doppler US diagnostic apparatus, C1-5 convex array probe (2.0–5.0 MHz) and LA523 linear array probe (4–13 MHz) were utilized. In our hospital, the operation requires the authorization of the hospital. All doctors who operate lung puncture are doctors with more than 10 years of interventional experience. The grouping is determined by experienced doctors according to the size and location of the lesion. The coaxial biopsy group adopted coaxial needle (17G * 11.8 cm) and disposable automatic puncture needle (BioPince,18G * 15 cm), while the conventional biopsy group used disposable semi-automatic puncture needle (SOMATEX,18G * 15 cm).

**Figure 1:**
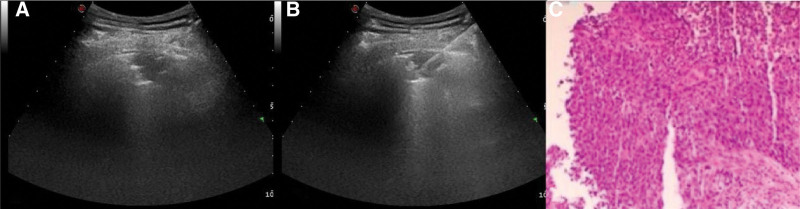
(A) The size of the mass was 26.7 mm * 15.7 mm, on the dorsal side of the lower lobe of the right lung, the coaxial needle was punctured to the edge of the lesion; (B) The automatic puncture needle was then inserted through the coaxial needle sheath for the medical removal of the tissue; (C) Pathology suggests poorly differentiated squamous cell carcinoma of the lung.

(1) Preoperative preparation: preoperative routine examinations were arranged, including blood routine examination and coagulation function examination. US examination was carried out in accordance with the routine chest CT examination. On the basis of the position of the lesion, the US imaging indicators such as gain, depth, dynamic range and focus area, were adjusted to obtain the best imaging effect; and the following multi-section observation of the target lesion were made to measure the position and size of the lesion and investigate the internal echo, blood flow and the existence of large blood vessels around the lesion. The feasibility of the needle biopsy and the safety of the designed puncture route were preliminarily evaluated, and the puncture point was located on the body surface subsequently. All participants gave written informed consent.
(2) According to the location of pulmonary lesions, the proper decubitus (supine position, prone position or lateral position) was selected for the patient. Routine disinfection was conducted with drapes rolled out. 2% lidocaine was injected into the pleural surface for local infiltration anesthesia.
  Coaxial biopsy group: under the guidance of US, the coaxial needle was punctured to the edge of the lesion, the needle core was pulled out, and a certain length of the cutting groove was selected according to the size of the lesion. The automatic puncture needle was then inserted through the coaxial needle sheath for the medical removal of the tissue (Fig. 1). 1 ~ 3 tissue samples were fixed in 10% formalin solution and sent for inspection. According to the reference,^[9]^ it is considered that 3 coaxial needle passes might be optimal in the diagnosis of. In addition, the specific puncture needles shall be determined according to the patient’s tolerance and complications (such as bleeding, pleural reaction, etc).
  Conventional biopsy group: the disposable semi-automatic puncture needle was inserted into the intercostal space under the guidance of US. When the tip of the needle reached the edge of the mass, the puncture needle slot was pulled out and the patient was required to hold the breath at the same time. The puncture needle was triggered instantaneously once the optimal lesion image was obtained. The specimen was fixed in 10% formalin solution, and 1 ~ 3 samples were obtained based on the standard volume.
(3) After the puncture, bandage and compression were applied to stop the bleeding for 10 minutes. US assessment was performed to check for needle-tract hemorrhage and pneumothorax. Meanwhile, some extra attention was also paid to check for the occurrence of certain complications such as hemoptysis and pleural reaction.

### 2.3. Observation index and evaluation standard

The observation indices included the average puncture time, number of sampling, satisfaction rate of sampling, success rate of puncture and puncture complications. The average puncture time was defined by the following equation:


$$\begin{array}{l} averagepuncturetime=\frac{thetotalpuncturetime(i.e.fromthepointofthepunctureneedlepiercingtheskintotheendoftheentirepunctureprocess)}{thenumberofthepunctureneedles} \end{array}$$

(Note: the entire puncture process = puncturing stage + sampling stage)

The obtained tissue sample which was greater than 10 mm were classified as satisfactory samples. The standard of a successful puncture was to obtain a definite pathological diagnosis. A definite pathological diagnosis included: the pathological result was malignant tumor; the pathological result was a specific benign lesion such as inflammation, tuberculosis and fungus, and the related imaging lesions had significantly shrunk or disappeared after anti-inflammatory, anti-tuberculosis or anti-fungal treatment (follow-up visit for at least 6 months). The criteria for an unsuccessful puncture were that the obtained specimens failed to gain a clear pathological diagnosis, including: too little effective tissue was found; only nonspecific tissue such as lung tissue, lymphocytes, and degenerated and necrotic tissue was observed under the microscope.

### 2.4. Statistical analysis

*SPSS* 22.0 statistical software was used in this report to analyze the data. For the measurement data (which conformed to the normal distribution and were expressed in *x ± s*), the independent sample *t* test was adopted for the comparison between the 2 groups. For the counting data (represented by the number of cases [%]), the comparison between the 2 groups was performed by *χ*^2^ test or Fisher’s exact test. *P* values <.05 indicated statistical significance.

## 3. Results

### 3.1. Participant characteristics

A total of 157 patients (73 from the coaxial biopsy group and 84 from the conventional biopsy group) with peripheral pulmonary masses underwent US-guided biopsy. There was no significant difference in age, gender, maximum diameter of the lesion, depth of the anterior edge of the lesion, lesion location, probe scanning position and lesion size between the 2 groups (*P* > .05), as shown in Table 1.

**Table 1:** Comparison of general data between the 2 groups.

Parameter	Conventional biopsy group (n = 84)	Coaxial biopsy group (n = 73)	Statistical value	*P*
**Median age ± SD**	65.4 ± 10.0	68.1 ± 10.7	1.621	.107
**Gender**			0.911	.340
Male	60 (71.4)	57 (78.1)		
Female	24 (28.6)	16 (21.9)		
**Maximum diameter of lesion (mm**)	46.0 ± 19.7	40.9 ± 17.8	1.716	.088
**Lesion depth (mm**)	27.0 ± 4.4	25.9 ± 4.7	1.556	.122
**Lesion location**			1.879	.758
Upper lobe of right lung	28(33.3)	22(30.1)		
Middle lobe of right lung	3(3.6)	5(6.8)		
Lower lobe of right lung	17(20.2)	17(23.3)		
Upper lobe of left lung	16(19.0)	10(13.7)		
Lower lobe of left lung	20(23.8)	19(26.0)		
**Probe scanning position**			2.304	.136
Anterior chest wall	25(29.8)	30(41.1)		
Lateral chest wall	21(25.0)	14(19.2)		
Posterior chest wall	38(45.2)	29(39.7)		
**Lesion size classification**			3.345	.067
≤3 (cm)	25(29.8)	32 (43.8)		
>3 (cm)	59(70.2)	41(56.2)		

### 3.2. Pathological results

One hundred fifty-seven patients underwent puncture biopsy and 145 patients eventually obtained definite pathological results. The overall puncture success rate for the entire study was 92.4% (145/157), while the puncture success rates for the coaxial biopsy group and the conventional biopsy group were 97.3% (71/73) and 88.1% (74/84), respectively. The difference between the 2 groups was significant (*P* < .05). The histopathological results were displayed in Table 2. Sixty-two cases of malignant tumors and 9 cases of benign lesions were found in the coaxial biopsy group, with only 2 cases having failed to obtain definite pathological results; whereas 63 cases of malignant tumors and 11 cases of benign tumors were observed in the conventional biopsy group (other 10 cases without clear pathological results).

**Table 2:** Pathological results of lung puncture.

Parameter	Conventional biopsy group (n = 84)	Coaxial biopsy group (n = 73)	Total
Successful puncture	74	71^*^	145
Malignant			
Adenocarcinoma	34	33	67
Squamous cell carcinoma	22	18	40
Large cell carcinoma	2	3	5
Metastatic carcinoma	1	3	4
Adenosquamous carcinoma	1	0	1
Sarcomatoid carcinoma	1	0	1
Pleomorphic carcinoma	1	0	1
Small cell carcinoma	1	3	4
Lymphoma	0	1	1
Neuroendocrine carcinoma of lung	0	1	1
Benign			
Pneumonia	9	5	14
Abscess	1	3	4
Fungus	0	1	1
Solitary fibroma	1	0	1
Puncture failure	10	2	12

* *χ*^2^** = **4.648, *P = *.031.

### 3.3. Comparison of puncture biopsy between the 2 groups

There were 57 cases for peripheral pulmonary masses ≤3 cm, with 32 cases from the coaxial biopsy group and 25 cases from the conventional biopsy group. The average puncture time per needle in the coaxial biopsy group was 35.3 ± 4.7 seconds, which was shorter than 64.7 ± 8.7 seconds in the conventional biopsy group (*P* < .05). The number of sampling, sampling satisfaction rate and puncture success rate in the coaxial biopsy group were higher than those in the conventional biopsy group (*P* < .05). There was no significant difference in the complication rate between the 2 groups (*P* > .05), as shown in Table 3.

**Table 3:** Comparison of puncture success rate and complication rate between the 2 groups ≤3 cm.

Parameter	Conventional biopsy group (n = 25)	Coaxial biopsy group (n = 32)	Statistical value	*P*
Sampling times(freq)	2.2 ± 0.7	2.8 ± 0.6	3.068	.003
Average puncture time ± SD(s)	64.7 ± 8.7	35.3 ± 4.7	16.438	.000
Sampling result,n(%)			5.728	.017
Satisfied	15(60.0)	28(87.5)		
Dissatisfied	10 (40.0)	4(12.5)		
Puncture result, n(%)			4.776	.029
Success	17(68.0)	30(93.7)		
Fail	8 (32.0)	2 (6.3)		
Complications, n(%)			0.074	.786
Occurred	21(84.0)	26 (81.3)		
No occurred	4 (16.0)	6 (18.7)		

For peripheral pulmonary mass >3 cm, there were 100 cases collected (41 cases from the coaxial biopsy group and 59 cases from the conventional biopsy group). The average puncture time in the coaxial biopsy group was 31.3 ± 5.3 seconds, which was shorter than 47.3 ± 7.4 seconds in the conventional biopsy group (*P* < .05). There was no significant difference between the 2 groups in the number of sampling, the satisfaction rate of sampling, the success rate of puncture and the incidence of complications (*P* > .05), as demonstrated in Table 4.

**Table 4:** Comparison of puncture success rate and complication rate between the 2 groups ≤3 cm.

Parameter	Conventional biopsy group (n = 59)	Coaxial biopsy group (n = 41)	Statistical value	*P*
Sampling times (freq)	2.9 ± 0.3	3.0 ± 0.2	0.335	.738
Average puncture time ± SD (s)	47.3 ± 7.4	31.1 ± 5.3	11.937	.000
Sampling result, n (%)			1.192	.275
Satisfied	53(89.8)	40(97.6)		
Dissatisfied	6(10.2)	1(2.4)		
Puncture result, n (%)			0.216	.642
Success	57(96.6)	41(100.0)		
Fail	2(3.4)	0(0.0)		
Complications, n (%)			0.164	.685
Occurred	52(88.1)	35 (85.4)		
No occurred	7 (11.9)	6 (14.6)		

### 3.4. Complications of puncture biopsy

The complication rate in the coaxial biopsy group was 16.4% (12/73; 5 cases of needle-tract hemorrhage, 3 cases of pneumothorax, 3 cases of hemoptysis and 1 case of pleural reaction), whereas the incidence of complication in the conventional biopsy group was 13.1% (11/84; 2 cases of needle-tract hemorrhage, 2 cases of pneumothorax, 5 cases of hemoptysis and 2 cases of pleural reaction), as demonstrated in Table 5. Since the participants with pneumothorax had extremely mild or even no symptoms, no treatment was given by both groups. For the participants with needle-track hemorrhage, the coaxial biopsy group used coaxial needle sheath gelatin sponge strips to block off the bleeding, while the conventional biopsy group mainly adopted compression approach to stop the bleeding. With regard to the participants with hemoptysis, due to the small amount of bloody sputum they had, no specific treatment was given by both groups as well. In addition, the participants with pleural reaction were observed for 2 hours postoperatively, with their vital signs being monitored.

**Table 5:** Incidence of puncture complications in the 2 groups.

Parameter	Conventional biopsy group (n = 11)	Coaxial biopsy group (n = 12)	Total
Hemorrhage	2	5	7
Pneumothorax	2	3	5
Hemoptysis	5	3	8
Pleural reaction	2	1	3

## 4. Discussion

Due to the diversity of imaging features of pulmonary lesions and the lack of specificity in diagnosis, it is difficult to identify the lesions with high-risk malignant factors in imaging. The application of biopsy to obtain pathological results is an effective method to guide clinical diagnosis, treatment and follow-up.^[10]^ Timely acquisition of tumor tissue specimens is crucial for the early detection, early treatment, optimal treatment planning, as well as for the prognosis of patients. Unlike central lung tumors, whose pathological results are mainly obtained by fiberoptic bronchoscopy and CT-guided puncture biopsy,^[11]^ peripheral pulmonary masses mostly use US-guided percutaneous puncture biopsy to obtain specimens. Under the guidance of US, the entire puncture process (i.e., the puncturing stage + the sampling stage) can be monitored in real time,^[12]^ with the needle tip being displayed during the whole operation to avoid large blood vessels and important organs, thus improving the safety. Not only that, the US-guidance puncture biopsy can modify the puncture angles as well. The needle can be inserted obliquely,^[13]^ and enters the lesions covered by scapula, sternum and ribs through intercostal gap, obtaining satisfactory specimens. At present, according to the literature, the general success rate of conventional US-guided puncture biopsy for peripheral pulmonary masses is more than 80%,^[14]^ with some even as high as 95%.^[11]^ In this study, 157 patients underwent puncture biopsy, and 145 patients finally obtained clear pathological results. The puncture success rate was 92.4%. Therefore, US-guided puncture biopsy of peripheral pulmonary masses is undoubtedly a method with high puncture success rate.

As the medical equipment continues to develop, the coaxial puncture technique has been widely used in recent years because of its convenient operation. Studies have shown that the application of the easily handled coaxial puncture needle can avoid multiple insertions,^[15,16]^ and increase the success rate of puncture, specimen adequacy rate (i.e., sampling satisfactory rate) and diagnostic accuracy.^[17]^ The coaxial needle consists of a needle core and a needle sheath. It punctures to the edge of the lesion during the puncturing stage, makes use of the needle sheath to establish a biopsy channel and then draws materials for biopsy by the matched disposable automatic puncture needle during the sampling stage. This reduces the difficulty of sampling and the resistance of biopsy, thereby avoiding repeated needle insertion and effectively saving the puncture time.^[18,19]^ In this study, no matter in the > 3cm masse scenario or the ≤3cm masses scenario, the coaxial biopsy group had shorter average puncture time than the conventional biopsy group (*P* < .05). This is because the puncture mechanisms for conventional biopsy and coaxial biopsy are different. In the conventional biopsy group, each time of puncture had to be performed in real time under the guidance of US, and the needle operated would pass through skin, subcutaneous tissue, muscle and intercostal space every time, which increased the difficulty in puncturing and prolonged the puncture time. The small lesions (≤3 cm), which had smaller contact areas with the chest wall and narrower effective puncture channels,^[20]^ required higher techniques for puncture. For such smaller lesions, our coaxial biopsy group took full advantage of the coaxial needle sheath channel to complete multiple sample collections each time, reducing the puncture difficulty comparatively.

With the development of medicine, simple benign and malignant diagnosis can no longer meet the demands of clinical diagnosis and treatment. Molecular typing based on genetic characteristics has brought the lung cancer treatment into the era of individualized molecular targeted therapy. In the era of precision treatment, the histopathological classification, immunohistochemical investigation and molecular pathological examination of lesions all require a large number of samples.^[21]^ For small lesions of a relatively narrow effective puncture channel, conventional biopsy has certain difficulty in puncturing, and often results in small amount of satisfactory specimens. This is normally because, after the first sample collection, the small lesions can be easily obscured by minor amounts of escaping gases,^[22,23]^ which means no further puncture is possible to obtain satisfactory specimens. Meanwhile, coaxial biopsy can draw materials again and again through the coaxial needle sheath and complete the entire puncture process on the basis of the size and the free boundary adjustment of the lesion. This helps to achieve the goal of ’one piercing, multiple sampling from different locations and different angles’, and ensure the obtainment of effective pathological tissue and the integrity of specimens, thus improving the diagnostic efficiency.^[24,25]^ In this study, for the masses ≤3 cm, the coaxial biopsy group had more samples being taken during the operation than the conventional biopsy group, and outperformed the conventional group in sampling satisfaction rate and puncture success rate (*P* < .05). The puncture success rate of coaxial biopsy group in the masses ≤3 cm was approximately 93.7%, which was consistent with the success rate for small masses reported in many literature.^[14,26]^

US-guided percutaneous lung biopsy causes certain complications.^[27]^ The common complications normally are needle-track hemorrhage, pneumothorax, hemoptysis and pleural reaction.^[28]^ In this study, the overall complication rate of US-guided lung biopsy was 14.6%, which was consistent with those reported in the literature.^[29]^ Meanwhile, the complication rate of coaxial biopsy group was 16.4%, and that of conventional biopsy group was 13.1%. Regardless of the size of masses (>3 cm or ≤3 cm), the statistical differences in the complication rate were not significant. It is worth mentioning that the coaxial biopsy group were prone to having needle-track hemorrhage after operations, however, the application of gelatin sponge strip or the injection of a small amount of hemocoagulase through the coaxial needle sheath was adopted and proven effective for local hemostasis.

In summary, our study highlighted the distinct advantages of US-guided biopsy of peripheral pulmonary masses (being real-time, safe, easy to operate and of high success rate), and demonstrated that US-guided coaxial puncture biopsy outperformed the conventional puncture biopsy with shorter average puncture time, more numbers of sampling and better sampling satisfaction rate and puncture success rate (especially for small lesions). Further studies are warranted to provided more useful insights for the diagnosis, differential diagnosis and individualized accurate treatment of lesions.

## Author contributions

**Conceptualization:** Shuyi Lv.

**Data curation:** Meiwu Zhang, Baisong Zhang.

**Investigation:** Xiaoxiang Fan.

**Resources:** Jiazhen Zhu, Zhenhua Yang.

**Supervision:** Yan Zhang.

**Writing – original draft:** Meiwu Zhang.

**Writing – review & editing:** Shuyi Lv, Xiaoxiang Fan, Jiazhen Zhu, Baisong Zhang, Zhenhua Yang.
